# Overview of the 37th MaxEnt

**DOI:** 10.3390/e20090694

**Published:** 2018-09-11

**Authors:** Teresa C. M. Dias, Marcio A. Diniz, Carlos A. de B. Pereira, Adriano Polpo

**Affiliations:** 1 Department of Statistics, Federal University of Sao Carlos, Sao Carlos 13565-905, Brazil; 2 Department of Statistics, University of Sao Paulo, Sao Paulo 05508-090, Brazil

**Keywords:** maximum entropy, bayesian methods, workshop

## Abstract

The 37th edition of MaxEnt was held in Brazil, hosting several distinguished researchers and students. The workshop offered four tutorials, nine invited talks, twenty four oral presentations and twenty seven poster presentations. All submissions received their first choice between oral and poster presentations. The event held a celebration to Julio Stern’s 60th anniversary and awarded two prizes to young researchers. As customary, the workshop had one free afternoon, in which participants visited the city’s surroundings and experienced Brazilian food and traditions.

## 1. The 37th MaxEnt

The 37th MaxEnt (http://www.gis.des.ufscar.br/meetings/2017maxent/) was happened in Jarinu, SP, in 9–14 July 2017 and the event was aimed at: strengthen research and present contributions in all aspects in Bayesian methods and maximum entropy, as well as extend its application in problems in different scientific communities and in areas such as astronomy, reliability, cosmology, econometric, engineering, quantum mechanics, stochastic processes, survival, etc.; provide an environment in which Brazilian and international researchers could interacting, presenting recent developments and discussing related issues; allow Brazilian graduate students to have contact with senior researchers to discuss their work and to start possible contacts for future doctorate degree and post-doctorate projects.

The MaxEnt was organized by the Group of Inductive Statistics (GIS-UFSCar, http://www.gis.des.ufscar.br/). The Conference Committee Chair were: Adriano Polpo (UFSCar) and Julio M. Stern (IME-USP). The members of the Organizing Committee were: Carlos Alberto de Braganca Pereira (IME-USP), Francisco Louzada Neto (ICMC-USP), Hellinton H. Takada (Itau Asset Management), Juliana Cobre (ICMC-USP), Katiane Conceicao (ICMC-USP), Marcio Alves Diniz (UFSCar), Nestor Caticha (IF-USP), Rafael Bassi Stern (UFSCar), Rafael Izbicki (UFSCar), Teresa Cristina Martins Dias (UFSCar) and Victor Fossaluza (IME-USP).

The members of the Scientific Committee were: Adriano Polpo (UFSCar), Ali Mohammad-Djafari (CNRS), Ariel Caticha (University at Albany), Carlos Alberto de Braganca Pereira (IME-USP), Francisco Louzada Neto (ICMC-USP), Hellinton H. Takada (Itau Asset Management), Julian Center (Autonomous Exploration Inc.), Julio Stern (IME-USP), Kevin Knuth (University at Albany), Paul M. Goggans (University of Mississippi), Rafael Izbicki (UFSCar) and Robert Niven (UNSW).

The members of the Executive Committee were: Luana A. Takahashi, Rita Volckov and Sylvia Regina A. Takahashi.

The workshop included the following invited speakers: Ariel Caticha (University at Albany) Flavio B. Goncalves (UFMG), Udo von Toussaint (Max-Planck-Institut fuer Plasmaphysik), Karim Anaya-Izquierdo (University of Bath), Estevam R. Hruschka Jr. (UFSCar), John Skilling (Maximum Entropy Data Consultants), Thais Fonseca (UFRJ), Rubens Sampaio (PUC-RJ) and Kevin Knuth (University at Albany).

The four tutorials were presented by the following persons: Adriano Polpo (UFSCar), Ali Mohammad- Djafari (Centre National de la Recherche Scientifique), Hellinton H. Takada (Itau Asset Management) and Rafael B. Stern (UFSCar).

We emphasize the fact that the invited talks, tutorials and oral sessions were filmed. They are available at https://www.youtube.com/channel/UCvBinMGiKRNRo8g8F0fvjMg. This is the visibility of the work of our community, which we all wish to have.

## 2. Tutorials and Talks

This edition of MaxEnt offered four tutorials presented here in chronological order. The tutorial, by Hellinton Takada, *Methods for portfolio allocation* presented the main methodologies for portfolio allocation, focusing on computational optimization and statistical modeling. Agatha Rodrigues, Adriano Polpo and Carlos Pereira in *Reliability estimation in coherent systems* discussed the parametric and nonparametric estimation of the reliability function of coherent systems (series-parallel and parallel-series). Systems with masked data were also discussed. *A model of evolution for languages*, by Rafael B. Stern, presented a probability model for the evolution of languages and discussed advances in Sequential Monte Carlo to obtain the posterior for this model. Ali Mohammad-Djafari in *Approximate Bayesian Computation tools for large scale inverse problems and hierarchical models for big Data* discussed applications in which observations are modeled by means of an hierarchical model with high dimensional hidden variables. Since in such models it is intractable to compute the likelihood function, the tutorial discussed how to obtain the posterior distribution by using Approximate Bayesian Computation methods.

The workshop also held nine invited talks, which are presented in chronological order. Ariel Caticha discussed the derivation of dynamical laws, and in particular, of quantum theory as an application of entropic methods of inference. Flavio Goncalves proposed a hidden Markov mixture model for the analysis of gene expression measurements mapped to chromosome locations. Udo Toussaint described the use of Gaussian processes and developed (parallelizable) batch processing approaches for the propagation of uncertainty in computational models. Karim Anaya-Isquierdo introduced a survival analysis methodology based on the cumulative hazard function which generalizes models such as those of proportional hazards and accelerated failure time. Estevam Hruschka presented key concepts from NELL (Never-Ending Language Learner), which automatically retrieves semantic relations between words from the Internet. John Skilling presented the “Quantum Theory is not weird” talk. Thais Fonseca presented a new class of spatio-temporal models that allows kurtosis to vary spatially. Rubens Sampaio constructed a parametric model to analyze data from a dry-friction oscillator. Kevin Knuth presented the EXONEST Exoplanetary Explorer, which is a Bayesian exoplanet inference engine based on nested sampling. This engine was used to analyze Kepler and CoRoT data.

## 3. Entropy Special Issue and Conference Proceedings

This special issue of Entropy (http://www.mdpi.com/journal/entropy/special_issues/maxent17) was focused on all aspects of probabilistic inference, including applications, methodology and foundations.The conference proceedings [1] had twenty eight articles published. The issue is composed of nine articles, which are briefly discussed below in the order of their publication.

*EXONEST: The Bayesian Exoplanetary Explorer* [2] presented a Bayesian exoplanet inference engine based on nested sampling and originally designed to analyze archived Kepler Space Telescope and CoRoT (Convection Rotation et Transits planétaires) exoplanet mission data. The EXONEST package currently accommodates plug-and-play models which allow for both circular and eccentric orbits in conjunction with photometric effects, such as the primary transit and secondary eclipse, reflected light, thermal emissions, ellipsoidal variations, Doppler beaming and superrotation. The paper also discussed new efforts to include more subtle photometric effects involving reflected and refracted light.

*L1-minimization Algorithm for Bayesian Online Compressed Sensing* [3] proposed a Bayesian online reconstruction algorithm for sparse signals based on Compressed Sensing and inspired by L1-regularization schemes. It showed that, even when prior knowledge about the signal’s generation is limited, reconstruction is possible by introduction of a Laplace prior and of an extra Kullback–Leibler divergence minimization step for hyper-parameter learning.

*Exact Renormalization Groups as a Form of Entropic Dynamics* [4] discussed Renormalization Groups (RG), a set of methods that have been instrumental in tackling problems involving an infinite number of degrees of freedom, such as, quantum field theory and critical phenomena. The paper showed that exact RG equations are functional diffusion equations, which can be derived using entropic methods. The RG model’s flow was then described as a form of entropic dynamics of field configurations. In this approach the RG transformations receive a purely inferential interpretation that establishes a clear link to information theory.

*Classical-Equivalent Bayesian Portfolio Optimization for Electricity Generation Planning* [5] discussed a method for allocating electricity generation among several available technologies, which can be solved as a mean-variance optimization problem. While traditional approaches assumed that the mean and covariances of the costs are known, the paper proposed a Bayesian approach which attributes a prior to these unknown quantities. Both improper and proper prior distributions were used and shown to compare well to the traditional method.

*A Sequential Algorithm for Signal Segmentation* [6] presented an application of event detection in noisy signals. The paper analyzed 15 min samples of acoustic signals and builds an unsupervised method for finding sections or segments of the signal in which events of interest are likely to have occurred. The Bayesian method is based on a sequential algorithm that is based solely on the assumption that each event alters the energy of the signal.

*Feature Selection Based on the Local Lift Dependence Scale* [7] proposed a method for feature selection that is based on minimizing a cost function over an estimate of the observables’ joint distribution. While previous approaches perform a search over a Boolean lattice of feature sets (BLFS), the proposed method searched over a collection of Boolean lattices of ordered pairs (CBLOP). As a result, the method found not only the features that are most related to a given variable, but also explained which values of these features are most related to each value of the variable.

*Categorical Data Analysis Using a Skewed Weibull Regression Model* [8] showed a partition scheme to derive a multinomial regression model, under binary link functions. Based on the distribution function of a random variable with Weibull distribution, they proposed a new skewed link function that has as limit case the most commonly used models (logit, probit and complementary log-log). The estimation procedure was presented under frequentist and Bayesian paradigms. The proposed model was compared to other skewed link function in two data sets.

*Estimating Multivariate Discrete Distributions Using Bernstein Copulas* [9] presented a procedure for estimation of joint discrete distributions. Under nonparametric approach, the authors proposed a method, based on Bernstein polynomials copula, to estimate bivariate discrete distributions, both the marginals and the joint distribution. Also, they compared with a alternative method by simulated samples and presented an application.

*Global Optimization Employing Gaussian Process-Based Bayesian Surrogates* [10] described a method for global optimization based on Gaussian processes. The authors discussed the problem that a simulation process is time consuming. Under Bayesian paradigm they proposed surrogate model function adjusted self-consistently via hyperparameters to represent the data. This surrogate model is based on a Gaussian Process, where they focused in the expectation values to represent the data. They advocated that this procedure is a shortcut to find the maximum, solving the global optimization problem.

## 4. Celebration and Awards

The 37th MaxEnt held a special celebration for Julio Stern’s 60th anniversary. Julio encouraged and mentored many students in the development of original research, totalizing twenty two graduate student orientations until now. His scientific interests encompass a variety of topics, such as, statistical theory, optimization, logic, epistemology, philosophy of science and operational research. During celebration there were speeches from several of Julio’s colleagues and friends, such as Marcelo Lauretto, Celma Ribeiro, Carlos Humes, Marco Gubitoso and Stephen Vavasis.

The workshop also awarded two young researchers for the “Young Author Best Paper Entropy Award”. The MaxEnt 2017 scientific committee has selected StevenWaldrip [11], and the local award committee, with Carlos Pereira as chair, selected Roberta Lima [12].

## 5. Final Comments

The main objective this workshop was discussion about the Bayesian computational techniques as Monte Carlo Markov Chain, as well as approximate inferential methods, questions about foundation of probability and information theory. Also, present and discuss new applications of inference foundations of physical theories. The good level of the event had excellent talks given by researchers of international projection, whose works are part of the current scenario of applied mathematics and statistics.

The workshop had a total of seventy participants, belonging to these institutions: *Australia*: The University of New South Wales at Canberra; *Belgium*: Ghent University; *Brazil*: EACH-USP, ICMC-USP, IF-USP, IME-USP, Itau Asset Management, PUC-Rio, Serasa Experian SA, UEM, UFF, UFMG, UFRJ, UFSCar, UFU, UNICAMP; *France*: CentraleSupelec, CU Boulder; *Germany*: Max-Planck Institut fur Plasmaphysik, MPI-CBG; *New Zealand*: University of Auckland; *USA*: Hobart and William Smith Colleges, University at Albany, University of Mississippi, University of Pennsylvania; *United Kingdom*: Maximum Entropy Data Consultants, Oxford, University of Bath.

It is worth noting the significant participation of students (twenty nine), most presenting their works orally or in posters. This is an important indicator of a promising future for the areas covered by the event. An evaluation was carried out with the participants. In view of the evaluations the event was considered excellent and that it fully reached the proposed objectives.

## References

[1] Polpo A., Stern J., Louzada F., Izbicki R., Takada H. (2018). Bayesian Inference and Maximum Entropy Methods in Science and Engineering.

[2] Knuth K., Placek B., Angerhausen D., Carter J., D’Angelo B., Gai A., Carado B. (2017). EXONEST: The Bayesian Exoplanetary Explorer. Entropy.

[3] Rossi P., Vicente R. (2017). L1-Minimization Algorithm for Bayesian Online Compressed Sensing. Entropy.

[4] Pessoa P., Caticha A. (2018). Exact Renormalization Groups as a Form of Entropic Dynamics. Entropy.

[5] Takada H., Stern J., Costa O., Ribeiro C. (2018). Classical-Equivalent Bayesian Portfolio Optimization for Electricity Generation Planning. Entropy.

[6] Hubert P., Padovese L., Stern J. (2018). A Sequential Algorithm for Signal Segmentation. Entropy.

[7] Marcondes D., Simonis A., Barrera J. (2018). Feature Selection based on the Local Lift Dependence Scale. Entropy.

[8] Caron R., Sinha D., Dey D., Polpo A. (2018). Categorical Data Analysis Using a Skewed Weibull Regression Model. Entropy.

[9] Fossaluza V., Esteves L.G., de Pereira C.A.B. (2018). Estimating Multivariate Discrete Distributions Using Bernstein Copulas. Entropy.

[10] Preuss R., Toussaint U. (2018). Global Optimization Employing Gaussian Process-Based Bayesian Surrogates. Entropy.

[11] Lima R., Sampaio R., Polpo A., Stern J., Louzada F., Izbicki R., Takada H. (2018). Uncertainty Quantification and Cumulative Distribution Function: How are they Related. Bayesian Inference and Maximum Entropy Methods in Science and Engineering.

[12] Waldrip S., Niven R., Polpo A., Stern J., Louzada F., Izbicki R., Takada H. (2018). Bayesian and Maximum Entropy Analyses of Flow Networks with Non-Gaussian Priors and Soft Constraints. Bayesian Inference and Maximum Entropy Methods in Science and Engineering.

